# Application of Metabolomics in the Study of Starvation-Induced Autophagy in *Saccharomyces cerevisiae*: A Scoping Review

**DOI:** 10.3390/jof7110987

**Published:** 2021-11-19

**Authors:** Muhammad Luqman Nasaruddin, Khaizurin Tajul Arifin

**Affiliations:** Department of Biochemistry, Faculty of Medicine, National University of Malaysia Jalan Yaacob Latif, Bandar Tun Razak, Cheras, Kuala Lumpur 56000, Malaysia; mlnasaruddin@ukm.edu.my

**Keywords:** autophagy, starvation, metabolomic, metabolites, *Saccharomyces cerevisiae*, yeast, nutritional stress, nitrogen starvation, glucose starvation, self-degradation

## Abstract

This scoping review is aimed at the application of the metabolomics platform to dissect key metabolites and their intermediates to observe the regulatory mechanisms of starvation-induced autophagy in *Saccharomyces cerevisiae*. Four research papers were shortlisted in this review following the inclusion and exclusion criteria. We observed a commonly shared pathway undertaken by *S. cerevisiae* under nutritional stress. Targeted and untargeted metabolomics was applied in either of these studies using varying platforms resulting in the annotation of several different observable metabolites. We saw a commonly shared pathway undertaken by *S. cerevisiae* under nutritional stress. Following nitrogen starvation, the concentration of cellular nucleosides was altered as a result of autophagic RNA degradation. Additionally, it is also found that autophagy replenishes amino acid pools to sustain macromolecule synthesis. Furthermore, in glucose starvation, nucleosides were broken down into carbonaceous metabolites that are being funneled into the non-oxidative pentose phosphate pathway. The ribose salvage allows for the survival of starved yeast. Moreover, acute glucose starvation showed autophagy to be involved in maintaining ATP/energy levels. We highlighted the practicality of metabolomics as a tool to better understand the underlying mechanisms involved to maintain homeostasis by recycling degradative products to ensure the survival of *S. cerevisiae* under starvation. The application of metabolomics has extended the scope of autophagy and provided newer intervention targets against cancer as well as neurodegenerative diseases in which autophagy is implicated.

## 1. Introduction

The maintenance and preservation of life are constantly under threat of fluctuations or limited availability of nutrient supply [1]. Fasting, an act of refraining oneself from drinking and eating within a specified amount of time, either voluntarily or obligatorily [2,3], showed beneficial outcomes. In humans, fasting and fasting-mimicking diets (FMDs) give many beneficial effects, such as improving cognitive function in older adults [4], lowering blood pressure, reducing total body fat, weight, and trunk, and decreasing insulin-like growth factors 1 (IGF-1) [5]. In fruit flies [6] and cell cultures [7], fasting extends lifespan, while in mice, it up-regulates the longevity Sirt1 gene [8] and preserves skeletal muscle with aging [9].

One of the effects of fasting or nutrient deprivation is autophagy [10]. Autophagy is a self-degradation process by which the cell consumes its damaged or mutant proteins and organelles to maintain cellular homeostasis [11]. Cells have evolved to elaborate metabolic responses to nutritional stressors that would allow them to break down and recycle endogenous macromolecules and reuse them as building blocks for the synthesis of other macromolecules to maintain the production of energy [12]. The three tracks of autophagy are macroautophagy, microautophagy, and chaperone-mediated autophagy (CMA). In macroautophagy, autophagosome engulfs targeted cargo and merge with lysosomes, whereby the lysosomal enzymes proceed to degrade the content [13]. In contrast, microautophagy involves direct lysosomal invagination of the cargo [14]. In CMA, the process of transporting the chaperone-targeted protein into the lysosome is aided by lysosomal membrane-associated protein [15]. Impaired autophagy has been linked with the accumulation of damaged mitochondria [16], West syndrome [17], inducing parenteral nutrition-associated lung injury [18], and the worsening of Alzheimer’s disease [19]. In unicellular organisms such as the yeast *Saccharomyces cerevisiae*, fasting is simulated in research by nutrient deprivation (or nutrient starvation) [20] which also resulted in extended lifespan [21].

Baker’s yeast *Saccharomyces cerevisiae* has been used as research models for diseases such as neurodegenerative disorders [22], aging [23], lifespan [24] oxidative stress [25], and autophagy [26]. The genes in *S. cerevisiae* are highly homologous to those in humans [27,28], and the simplicity of manipulating the genes [14,29,30] and nutrition [31,32] of this short-lived yeast make it a favorable eukaryotic representative. Autophagy defective mutant, *atg1* was first discovered in *S. cerevisiae*, and through this led to the discoveries of other ATG related genes that are involved in the autophagy following starvation [33].

Autophagosome biogenesis in *S. cerevisiae* involves at least 13 vital ATG genes that encode the respective Atg proteins. Atg13 has a vital role in initiating the process, by stimulating Atg1, a serine/threonine kinase in the formation of pre-autophagosomal structure [34]. *Atg1* and *Atg2* encode Atg1 (serine/threonine kinase) and Atg2 proteins, respectively. Both are necessary for autophagy vesicle production and the cytoplasm-to-vacuole targeting (Cvt) pathway [35]. Atg3, encoded by *Atg3*, is an enzyme that catalyzes the formation of Atg8-phosphatidylethanolamine conjugates, which is also mediated by Atg7, a vital step for Atg8 lipidation [36,37]. Atg4 is a protease that cleaves Atg8 to form autophagosomes and vesicles [36]. Atg5 conjugates with Atg12 (mediated by Atg10) [36] and Atg16 to bind to the membrane to efficiently promote Atg8 lipidation [36,38]. Atg6 (encoded by *Atg6*) is an essential subunit of phosphatidylinositol 3-kinase complexes I and II, required for the localization of Atg8 and Atg5-Atg12-Atg16 complex to the phagosome assembly site [39]. Another protein that facilitates the attachment of Atg12 to Atg5p and Atg8 is Atg7, which is encoded by *Atg7* [36]. At the membrane, a transmembrane protein Atg9 plays a big role in autophagic vesicles biogenesis [35]. Atg11 acts as a protein scaffold directing the receptor-bound cargo to the phagophore assembly sites [40].

Metabolites or small molecules entities (metabolomes) (<1 kDa) represent the downstream products of the complex interactions that define biological processes and functions expressed by the genes (genomes), transcripts (transcriptome), and proteins (proteomes) [41,42]. They are highly influenced by the interaction of both genetic as well as the external and/or internal environments, and as such, the flux that brought about the changes of their levels offers a very close measure of the organisms’ physiology [43,44]. Due to this, the study of metabolites has become an important tool in predicting and/or profiling key metabolic biomarkers involved in maintaining homeostasis or dealing with stressful conditions. Metabolomics is a study that systematically identifies and quantifies these small molecules through high-throughput detection methods within a specific time frame [43]. The study of yeast metabolites has only been widely conducted through traditional analytical means [45]. Therefore, a comprehensive view of the organism’s metabolome is still inadequate. As mentioned previously the process or induction of autophagy is tightly dependent on the cellular stress status. As such, autophagy-related metabolomes will be subjected to change according to the nature of the stresses occurring in the cells [46,47]. This scoping review aims to observe the application of metabolomics on how it offers a deeper and dynamic understanding of the metabolic or physiological function of autophagy in *S. cerevisiae* following nutrient starvation. We hope to identify gaps in the literature whilst providing suggestions for future consideration.

## 2. Materials and Methods

A systematic search was undertaken to map out pertinent articles for this scoping review. Full-text, peer-reviewed English articles were searched through multiple electronic databases including, Scopus, Pubmed, and Web of Science as early as 1960 to 2021. Search terms together with the Boolean operators AND and OR were as follows: “saccharomyces cerevisiae” OR “yeast” OR “baker’s yeast” AND “metabolom*” AND “autophagy”. The inclusion criteria for this study were that: (1) the study must focus on the subject of autophagy, (2) metabolomics has to be incorporated into the study (either targeted, untargeted, or the combination of both), and lastly, (3) starvation as a method to induce autophagy is mentioned as part of the study. In contrast, the exclusion criteria for the study were (1) studies that involve intervention (of any kind) to the yeast, and (2) studies that were conducted on humans, animals, or any kind of yeast besides *S. cerevisiae*. Collated titles and abstracts from the multiple databases were initially screened and independently critiqued by both authors. The articles then were shortlisted for eligibility upon reaching a consensus following the inclusion and exclusion criteria specified at the start of the study.

## 3. Results

**Figure 1:**
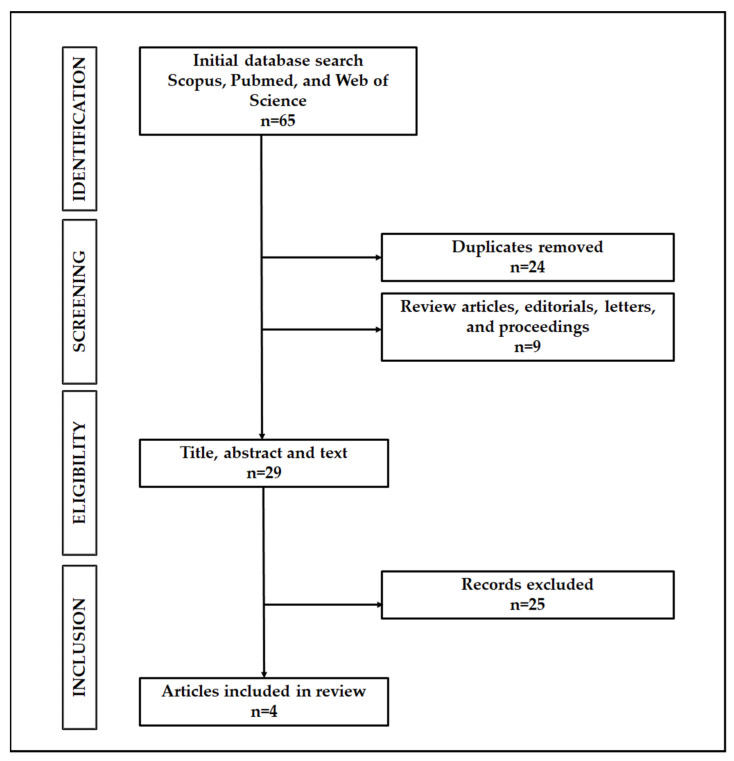
Search strategy result.

The literature search has identified a total of 65 papers (Figure 1).

In total, 24 papers were found to be duplicates, and nine other papers were review articles, editorials, letters, and proceedings. These were subsequently removed from the list. The remaining articles were then screened for eligibility based on the inclusion and exclusion criteria. Four articles met the inclusion criteria and were included in the current review (See Table 1 and Table 2 for a comprehensive summary).

At least three different nutrient-starved conditions were reported in the review. Nitrogen starvation was conducted for three experiments [14,48,49], while three other studies reported findings for glucose starvation experiments [14,32,49], and only one experiment looked into the metabolite changes under phosphate starvation (Table 1) [48]. Targeted and or untargeted metabolomics was applied, and metabolites of interest were determined through tandem LC-MS/MS or flow-injection Time-Of-Flight-MS methodology (Table 1). We identified a considerable overlap in terms of the metabolites that were being studied. Products of RNA and nucleotide degradation were being measured in two studies under contrasting starvation conditions (Table 2) [48,49]. One study looked into the effects of autophagy on the synthesis of specific amino acids [14] while the remaining one looked into the effects of autophagy on ATP levels as well as the changes of various lipid metabolites during starvation (Table 2) [32].

Under nitrogen starvation conditions, in one study, the levels of nucleosides (adenosine, guanosine, and cytidine) were found to be transiently increased in wild-type yeasts than *atg2*Δ strain following 2 h of starvation (Table 2) [49]. However, in the same study, for inosine and uridine, a steadily increasing level of these two nucleosides was observed in wild-type than *atg2*Δ strain over time (Table 2) [49]. Following this, the study also demonstrated a transient increase in the concentration of intracellular nucleobases; guanine, xanthine, and uracil in wild-type as opposed to *atg2*Δ strain in the cytoplasm (Table 2). Similar to inosine and uridine, hypoxanthine and uracil exhibited a steady increase concentration in wild-type than *atg2*Δ cells (Table 2) [49]. The autophagic degradation of RNA in the vacuole was made possible with the assistance of Rny1, a vacuolar RNase as high levels of 3′-nucleoside monophosphates (NMPs) in wild-type were observed as compared to *atg2*Δ and *rny1*Δ cells (Table 2) [49]. The generated nucleotides were catabolized further into nucleosides as described above with the assistance of a non-specific phosphatase Pho8. The study demonstrated an increase in the levels of nucleosides in wild-type than in *atg2*Δ and *pho8*Δ cells (Table 2). As nucleosides are then transported to the cytoplasm, they are further degraded by two nucleosidases, Pnp1 (a purine nucleoside phosphorylase that acts on guanosine and inosine) and Urh1 (a pyrimidine nucleoside-specific hydrolase that converts cytidine and uridine into cytosine and uracil) (Table 2). The study demonstrated an increase in the intracellular levels of nucleosides and nucleobases in wild-type than *atg2*Δ cells. These were then excreted extracellularly especially for inosine, guanosine, cytidine, uridine, xanthine, and uracil (Table 2) [49]. Interestingly, under the same starvation condition, Xu and colleagues saw a similar increasing trend of nucleoside and nucleobase levels in wild-type as compared to *atg7*Δ strain after 3 h of starvation [48]. In contrast to the previous study, the ratio of deleted strain over Wildtype showed decreasing levels of inosine, guanosine, uridine, cytidine, hypoxanthine, guanine, and uracil. While adenosine and guanine remained unchanged (Table 2) [48]. The current study also detected a significant improvement of glutamine levels in wild-type as opposed to *atg7*Δ cells.

However, unlike the transient levels of nucleosides and nucleobases demonstrated under nitrogen starvation conditions, autophagy causes a steady increase of these molecules under carbon starvation. Autophagy in yeast that was grown in glucose/carbon-deprived media was shown to induce an increase in the levels of nucleosides as detected in wild-type in comparison to *atg2*Δ [49] and *atg7*Δ [48]. The induction of autophagy was made possible by AMP-activated protein kinase (SNF1) (active in the absence of glucose) and the inactivation of cyclic-AMP dependent protein kinase (PKA) (an inhibitor of autophagy, however, becomes inactivated upon glucose removal), where the levels of nucleosides and bases were found to be diminished in *bcy1*Δ (allows for activated PKA activity) and *snf1*Δ (lacks SNF1 activity) strain in comparison to wild-type (Table 2) [48]. Moreover, similar to the condition of nitrogen starvation, nucleosides are further degraded into nucleobases, and further broken down into sedoheptulose species by Pnp1 and Urh1 (Table 2) [48]. Wild-type cells were found to have high levels of sedoheptulose-7-phosphate (S7P) and ribose-5-phosphate (R5P) in comparison to *pnp1*Δ and *urh1*Δ strains (Table 2). These phosphate species were initially derived from ribose phosphates as a result of RNA degradation through the help of transketolase isozymes (TKL1/TKL2) that are responsible for the conversion of pentose phosphates into S7P. High levels of S7P and R5P were observed in wild-type cells as compared to *tkl1*Δ *and tkl2*Δ strains under the current starvation procedure (Table 2) [48].

Additionally, glucose starvation has resulted in the depletion of global intracellular metabolite pools in both wild-type as well as anabolic respiration-deficient cells, *cbp2*Δ. After 1 to 4 h of glucose starvation, metabolites including glycerol-phosphate, fructose bisphosphate, glucose-6-phosphate (G6P), acetyl-CoA, glutamate, and glutamine were found to be affected in both of these cells with G6P, acetyl-CoA, R5P, and glutamate were found to be completely depleted in *cbp2*Δ strains than wild-type (Table 2) [32]. Further exploration into the extracellular pools of metabolites namely amino acids demonstrated a rapid depletion of aspartate and methionine in yeast fed in glucose-deficient media as opposed to a glucose-rich environment (Table 2) [32]. The uptake of amino acids did not affect the levels of ATP following acute glucose starvation [32]. A subsequent look into the much shorter time frames (10–60 s) of starvation revealed a rapid and significant depletion of internal glucose levels in yeast in glucose-deprived media as opposed to glucose-fed media [32]. Additionally, yeast under the glucose-deprived media demonstrated rapid reduction of hexose phosphates, ribose phosphates, as well as pentose following in comparison to yeast in glucose-rich conditions (Table 2). Further work on the yeast’s lipidome demonstrated only specific lipids and lipid classes such as polyketides and dolichols were found to be affected during starvation (Table 2). µ-lipophagy, as mediated by *atg14*Δ strains, did not contribute to energy maintenance. Maintenance as noted in this study was done through B-oxidation as well as autophagy since double deletion of *POT1* (a thiolase that catalyzes the last step of B-oxidation) and *ATG2* genes resulted in reduced levels of intracellular ATP as compared to single-deletion mutants and more importantly to wild-type (Table 2).

Moreover, in a different study following a 6 h of nitrogen-starved condition, Liu and colleagues have demonstrated sustained macromolecule synthesis as induced by autophagy in wild-type as opposed to *atg1*Δ cells (Table 2). Amino acid levels were found to be preserved in wild-type as compared to *atg1*Δ strain, despite exhibiting comparable profiles in a previous nitrogen-fed environment [14]. The study also noted that autophagy promotes the synthesis of specific amino acids; namely, aspartate and glutamate as is it abundantly found in wild-type than *atg1*Δ strain (Table 2) [14]. Moreover, ammonium was found to be an important nitrogen source for glutamate and aspartate synthesis as the failure of its assimilation through the GOGAT pathway resulted in reduced levels observed in wild-type as compared to *gdh1*Δ, *gdh3*Δ, and *glt1*Δ cells (glutamate dehydrogenase and glutamate synthase; allows for ammonia assimilation processes to form glutamate). In addition, nitrogen-starved WT cells were found to utilize significantly higher levels of aspartate in contrast to the *atg1*Δ strain (Table 2) [14]. Aspartate was found to be incorporated more into proteins and nucleic acids.

Interestingly, the sole research paper that looked into the effects of autophagy on yeast undergoing phosphate starvation, showed no impairment of nucleosides accumulation in *atg7*Δ in comparison to wild-type [48].

## 4. Discussion

The current review summarizes the findings of four separate studies that utilized metabolomics on two different starvation conditions in yeast—glucose or nitrogen. The application of metabolomics platforms provided a fresh outlook of the dynamic changes as well as the context-specific state that are occurring in yeast under stressful situations. The platform offered a reliable characterization of metabolites that regulate the cellular processes of autophagy. As the rate of turnover of molecules in a given pathway can increase rather rapidly in comparison to changes occurring at the translational level or protein abundances, metabolites would likely be among the first to respond in the new environment. The application of metabolomics would then provide a much accurate depiction of the ongoings in a particular organism in response to particular stimuli [50].

Autophagy is a highly conserved intracellular process by which cells direct their components to the lysosome via autophagosomes for degradation or recycling of nutrients and organelles, to maintain homeostasis under normal and or otherwise undesirable conditions [51,52,53]. It is induced under nutritional stress [33,53] and starvation [54] and involves proteins that participated in the formation and function of autophagosomes [1,53]. Protein degradation via autophagy was confirmed in yeast cells following the isolation of *ATG* genes (autophagy-related genes) [26,33,55]. In total, 18 Atg proteins; Atg1-Atg10, Atg12-Atg14, Atg16-Atg18, Atg29, and Atg3, were identified as essential for starvation-induced none selective autophagy [1]. A sufficient supply of nutrients is vital for the overall maintenance and preservation of life. The survival and effective resumption of growth are crucial through the recycling and salvage of molecules until conditions are improved.

### 4.1. Nucleosides and Nucleobases

#### 4.1.1. Nitrogen Starvation

The application of mass spectrometry as reported in this review has led to the discovery of the temporal changes of intracellular RNA-derived metabolites following nitrogen starvation. Nitrogen starved yeast cells exhibited a transient increase in the relative levels of nucleosides followed by the increase in the levels of purine and pyrimidine nucleobases in the organism following starvation [48,49]. This was made possible through the breakdown and hydrolyzation of sequestered RNAs (3′NMPs) in the vacuole with the help of T2-type RNase and phosphatase; Rny1 and Pho8 [49]. Similar depletion of RNAs has recently been reported in *Ure2*Δ strains as compared to wild-type [56]. Deletion of the *URE2* gene induces a similar physiological state as nitrogen starvation and autophagy in yeast was consistent with the bulk-autophagy pathway [56].

To date, the question of whether that autophagy-induced RNA degradation occurs preferentially or non-selectively is yet to be fully understood [57]. While it was previously suggested that ribosomes are selectively degraded via ribophagy in a Ubp3 (deubiquitinase) -Bre5 (its co-factor) dependent manner [58], findings from Huang and colleagues reported here [49], have shown only temporal delay in RNA degradation for individual *Bre5*Δ and *Ubp3*Δ strains relative to wild-type cells. The deletion of these genes did not for the most part block autophagy-induced RNA breakdown as RNAs were still being delivered into the vacuoles [49]. This is key and interesting to note for future consideration as the need to identify and investigate whether the amount and/or types of RNA play a role in determining the type or preference specific autophagic pathways [59]. The previous study by Kraft and colleagues indicated the role of Ubp3 for the non-selective uptake of mature ribosomes for bulk autophagy and selective uptake of the 40S and 60S ribosomal subunits [58]. Further study on Ubp3 demonstrated its mediation on selective degradation of translation and RNA turnover factors under nitrogen-starved conditions [60]. A most recent study has reported that a subset of mRNAs that encodes for amino acid biosynthesis and ribosomal proteins were preferentially delivered to the vacuole by in rapamycin-induced autophagy in yeast for subsequent Rny-1 mediated degradation in the vacuole [57].

Our metabolomics review also reported the fate of RNA catabolism products namely purines and pyrimidines, as they were eventually excreted rather than salvaged by yeast cells under nitrogen starvation conditions [49]. While it is unclear as to this paradoxical endpoint, one recent study has pointed that the plausible reason for such occurrence could be to compensate for the short supply of available nucleobases [61]. The study has noted that the induction of autophagy following purine and pyrimidine starvation has resulted in the reuptake and salvage of extracellular nucleobases to rescue cells [61]. Additionally, it is also reported that following the drop in nucleotide pools, survival during starvation in autophagy-deficient Kras-driven lung tumor cells was fully rescued by glutamine, glutamate, or nucleosides [62].

#### 4.1.2. Carbon Starvation

Moreover, targeted metabolomics analyses have allowed for the elucidation of the nucleotide degradation pathways in glucose-starved yeast cells [48]. We saw a considerable overlap with regards to the autophagy-induced breakdown of RNA metabolites as seen in nitrogen starvation conditions. Starvation of either through glucose or nitrogen in yeast cells has caused an accumulation of nucleosides, nucleobases, as well as sedoheptulose species (only for glucose starvation). Autophagy in the study was found to be induced by the activation of SNF1, an AMP-activated protein kinase, as well as the inactivation of cyclic-AMP dependent protein kinase (PKA). The activities of these two molecules were confirmed in a much recent study by Adachi and colleagues [63] and are key for the induction of autophagy during glucose starvation. Phm8 as shown in the current study was found to be a crucial nucleotidase in yeast. Moreover, similar to nitrogen conditions, purines and pyrimidine were broken down with the help of Pnp1, and Urh1 proteins. *These proteins are essential in ribosome salvage as individual deletion of PNP1, URH1, and PHM8 exhibited a reduction in sedoheptulose species (S7P and SBP); key ribose-derived carbon species that are central in the conversion of ribose into glycolytic intermediates* [48]. In addition, long-term starvation in these mutant strains caused a decreased viability in comparison to wild-type, which shows that nucleotide degradation and ribose salvage are essential for survival under stress [48]. Ribose salvage during glucose starvation in yeast requires intact non-oxidative pentose-phosphate pathways and the increase in the levels of sedoheptulose species as the author suggested provides nutrient reserves of carbon and phosphate for the cells [48]. The previous study has indicated that autophagy is closely linked to the metabolic state of the cell [63]. Gene expression for the utilization of alternative carbon sources and respiration is strictly repressed in the presence of sufficient glucose [63]. This carbon catabolic repression negatively regulates autophagy under carbon starvation and is positively correlated with yeast’s respiratory metabolism [63].

### 4.2. Amino Acid

#### 4.2.1. Nitrogen Starvation

Interestingly, through mass spectrometry, it was also demonstrated that autophagy is a crucial stress response in maintaining the amino acid pools to sustain the survival of yeast [14,64,65]. In this review, studies have reported that nitrogen-starved yeast cells are capable of synthesizing amino acids at varying degrees with much emphasis on glutamate and aspartate [14]. Amassing glutamate is needed as it is a major cellular nitrogen donor that replenishes nitrogen levels to support macromolecule synthesis [14,66]. The study also found that ammonium from degraded proteins is an important source to assimilate via the GS-GOGAT pathway to synthesize glutamine, and subsequently glutamate and aspartate [14]. These nitrogen starved cells invested more glutamate in aspartate synthesis to produce derivative amino acids as well as providing the building blocks for proteins and nucleic acids [14]. Aspartate from this study was found to be significantly being incorporated into proteins [14]. Maintaining pools of amino acids as the author suggested, is key during starvation as it helps the cells to sustain protein synthesis [64], recycle and replenish nucleotide pools as previously mentioned, and is critical to complete its cell cycle [67]. A very recent study has shown that amino acid starvation inhibits autophagy in lipid-droplet deficient yeast [68]. Lipid droplets act as storage sites for neutral lipids. In response to nitrogen starvation, lipid droplets are required for autophagy to proceed and to maintain endoplasmic reticulum (ER) homeostasis [68].

#### 4.2.2. Glucose Starvation

With regards to glucose starvation, a global metabolome and lipidome profiling of yeast were successfully undertaken [32]. The study demonstrated that respiration is crucial for the survival of yeast cells, as the dramatic reduction of ATP was observed in *CBP2* deleted yeast as compared to isogenic wild-type [32]. The result was replicated in a previous study where an immediate drop in the levels of intracellular ATP was observed and persisted for 3 h following the onset of glucose starvation in wild-type yeast [63]. Further depletion of intracellular metabolite pools that are central in glycolysis and citric acid cycle were exhibited in this cell, with several metabolites including glutamate and acetyl-CoA were found to be completely depleted. While rapid depletion of extracellular amino acids, namely aspartate and methionine, was observed, it did not affect the ATP levels nor offer a long-term survival advantage to the cells in glucose and amino acid-deprived conditions as compared to glucose-only [32].

There is a need for a comprehensive look into the complex interplay between autophagy-derived amino acids and ATP/cellular energy requirements in health and diseased cells. A recent study on the mammary carcinoma cancer cell line has suggested that the maintenance of an intracellular pool of amino acids in MDAMB231 cells is critical for its homeostasis and preservation of ATP following acute amino acid starvation [69]. *Preferential utilization of autophagy-derived amino acids can help mitigate the reduced levels of ATP following amino acid starvation. However, this reliance on autophagy-produced substrates to maintain homeostasis is only transient.* [69]. Moreover, glucose starvation without supplementation of amino acids in gastric cancer cells causes inhibition of growth and cellular apoptosis [70]. By supplying non-essential amino acids to these cells, growth inhibition and apoptosis were rescued [70]. This shows that amino acids under the context of autophagy are crucial as an alternative energy source from other cells. Starvation-induced autophagy in the current study however has caused no decrease in the levels of ATP to the same extent as in *CBP2* deleted yeast implying an alternative pathway of generating short-term ATP during glucose starvation [32].

### 4.3. ATP and Lipids

#### Glucose Starvation

Further observation demonstrated that µ-lipophagy, the alternative degradative pathways that involve direct engulfment of lipid droplets into the yeast vacuole as opposed to the autophagosome-based lipophagy provides a long-term energy maintenance in glucose-starved [32]. The finding from this study is consistent with the previous report whereby acute glucose reduction in yeast led to the survival deficiency of the cells after 7 days [71]. AMP-activated protein kinase (AMPK) and ATG14 protein (a kinase) in yeast cells orchestrated lipophagy to extend yeast’s lifespan [71]. The author of the current work also suggested that AMPK may have been activated early during starvation [32]. In addition, it was also noted that the consumption of lipids through β-oxidation in the peroxisomes contributes to the intracellular levels of ATP within several hours of glucose starvation. Double deletion of β-oxidation and autophagy exhibited lower levels of intracellular ATP than the single-deletion mutants [32]. While it was previously argued that autophagy is dispensable in glucose-starved yeast cells and that vacuolar hydrolysis is key in replenishing cells with energy [72], the metabolome and lipidome analyses showed in the current study indicated that it is essential for cell survival and that ATP levels depend on autophagy within the first 24 h [32]. In addition, previous work has demonstrated alterations of lipid metabolism following starvation. The study on serum and amino acid-starved mouse embryonic fibroblast (MEF) demonstrated autophagy-induced changes of the cellular lipidome with significant alteration occurring to the free fatty acid, glycerophospholipid as well as sphingolipid metabolisms [73]. Lipids were consumed and protected these cells from death during starvation [73]. Cellular lipidome analysis in the current study exhibited no major changes in lipid levels between control and glucose starvation conditions [32]. While it was initially hypothesized that major remodeling of the membrane and or the liberation of lipids for energy synthesis would occur following starvation, the findings show that cellular membranes did not undergo significant changes and only specific classes of lipids were affected (polyketides and dolichols) [32].

### 4.4. General Application of Metabolomics in the Study of Starvation

In recent years, there is a rising interest in the application of metabolomics in the general study of starvation in healthy or diseased organisms other than yeasts. Fasting in humans or mice has resulted in major alterations of the plasma metabolome, with relatively minor changes occurring in the intracellular metabolome of circulating leukocytes [74]. Alteration of free fatty acids and/or different acylcarnitine species in the plasma as observed in the study is suggested to reflect the breakdown of lipids of endogenous storage under nutritional stress [75]. Moreover, in a different study, LC-MS analyses on mouse embryonic fibroblast cells (MEFs), had seen a similar alteration in amino acid, energy, carbohydrate, and lipid metabolism in response to nutrient stress [76]. Amino acid metabolism was found to be affected following acute starvation. Among the many affected pathways are glutamine and glutamate metabolism as well as aspartate and glutamate metabolism [76]. Induction of autophagy with subsequent upregulation of lipid metabolism offered protection to MEFs and delayed cell death [76]. Furthermore, subsequent GC-MS analyses on these cells provided additional altered metabolic pathways and products that were previously unaccounted for including valine, leucine, and isoleucine, as well new targets of glucose metabolism products following starvation [77]. In addition, in the study of cancer, a comprehensive metabolome analysis of glutamine-deprived cancer cells demonstrated an accumulation of phosphoethanolamine metabolite that protects the cancer cells through the downregulation of rate-limiting enzyme of phosphatidylethanolamine biosynthesis, PCYT2 [78]. Reduced expression of PCYT2 was correlated with decreased survival in cancer patients [78]. In a different study, metabolic pathways, including, amino acid, pyrimidine, glycerophospholipid metabolism, and the TCA cycle, were found to be most affected by arginine starvation in breast cancer cells xenograft models [79]. Mitochondrial dysfunction and aspartate exhaustion through arginine starvation allow cancer cells to be killed [79].

### 4.5. Study Limitation

Overall, the scientific papers that were compiled here in this review have demonstrated the practical application of metabolomics in the study of starvation-induced autophagy in *Saccharomyces cerevisiae*. The platform allows for the proper identification or annotation of metabolites in yeast that are involved in a particular pathway that regulates the different forms of starvation conditions. The limitation of this review is that it only focused on four research papers. Most if not all these papers are targeted studies on different forms of deletion strains. While these mutant strains are generally involved or partook in bulk autophagy (*ATG* gene for example), they serve different purposes. As such, a direct comparison between studies with regards to the metabolites involved must be taken with caution. However, it does not hide the fact that despite the differing starvation conditions and mutant strains, there exists an overlap of the metabolites involved suggesting a commonly shared pathway. Additionally, experimental protocols including extraction procedures and platforms that were applied to identify and measure these metabolites were found to be different across studies. This variation while can be a limitation, it, however, allows for the unique identification of metabolites. We believe that the effort of charting these metabolites and their intermediates has allowed us to observe the regulatory mechanism (the key enzymatic steps) involved in yeast’s survival while rectifying multiple different misannotations in the genome-scale yeast metabolic model [48]. Moreover, as previously mentioned, since metabolic pools are rapidly depleted within seconds [80] and the flux occurred at a much faster rate than transcriptional and or protein abundance changes [50], the metabolomics application reported in this review has successfully recorded metabolite alterations as an initial response following starvations that would have likely been missed on other platforms. The shifts provide a much accurate depiction of the ongoings in yeast in response to stressful environments.

The huge barrier when using yeasts as models is the actuality of dissimilarities of metabolic responses with humans. Although yeast synthesize numerous proteins orthologous to humans, yet some of its metabolism differs in many ways. One of them is the carbohydrate metabolism in *S. cerevisiae*, which occurs predominantly anaerobically in the presence of oxygen, depending on the source of carbon [81]. Furthermore, the catabolism of fatty acids via β-oxidation occurs exclusively in the peroxisome [82,83], as opposed to the mitochondria in humans [84]. Another example would be the fate of the cAMP/PKA signaling pathway. A similar fate as that to a human would be storing carbohydrates [85], but the deviated when the consequence leads to filamentous growth [86] and cell wall biosynthesis [87]. One way to overcome these differences and integrate the knowledge that the yeast can provide us is the use of humanized yeast models [88].

## 5. Conclusions

Through these studies, we have identified several gaps of which warrant further attention for future direction. As there exist several studies that explored nitrogen, carbon, and/or amino acid starvation-induced autophagy, there is yet a report on the application of metabolomics on other forms of starvation conditions including phosphorus and sulfur, or even metal ions. Moreover, looking into specific aspects of selective autophagy besides ribophagy, such as mitophagy, lipophagy, or proteophagy, should be further investigated with the application of metabolomics to help dissect the underlying mechanisms involved under nutritional stress. Additionally, metabolites that are involved in the transition from autophagy promoting cell health to programmed cell death remain elusive in starving yeast. We also hoped that the application of not just a single metabolomics platform, but cross omics technologies coupled with big-data analytics would help explain these gaps and providing new and vital targets in the finetune of autophagy processes following nutritional deprivations. The discovery of new metabolic (by)products, and by extension the pathways that are being regulated, provides potential intervention targets as predictive roles for studies such as cancer [30] as well as neurodegenerative diseases [52] of which autophagy is implicated.

## Figures and Tables

**Table 1:** The summary of data extracted from eligible articles. Abbreviations: POS (Positive Mode), NEG (Negative Mode), HPLC (High-performance liquid chromatography), MS (Mass Spectrometry).

Reference	Genetic Deletions	Starvation Conditions			Platform	Targeted/Untargeted	Mode
		Glucose	Phosphate	Nitrogen			
[48]	*atg7*Δ *bcy*Δ *snf1*Δ *sdt*Δ *pnp1*Δ *Urh1*Δ *pnp1*Δ*Urh1*Δ *pgm3*Δ *tkl1*Δ *tkl2*Δ *tal1*Δ *nqmn1*Δ *pfk1*Δ	√	√	√	Reverse-phase LC-MS/MS	Targeted	NEG
[49]	*atg2*Δ *atg7*Δ *atg17*Δ *pep4prb1*Δ *atg11*Δ *atg19*Δ *atg32*Δ *nvj1*Δ *pho8*Δ *rny1*Δ *bre5*Δ	√		√	Pentafluoro pentaphenyl LC-Triple Quadrupole-MS Reverse-phase LC-Triple Quadrupole-MS	Targeted	POS and NEG
[32]	*cbp2*Δ *Atg14*Δ *pot1*Δ *atg2*Δ*pot1*Δ	√			Flow injection (FIA) Quadrupole TOF	Untargeted	NEG
[14]	*atg1*Δ *atg6*Δ *atg8*Δ *atg9*Δ *atg15*Δ *ypt7*Δ *pep4prb1*Δ *atg19*Δ *atg32*Δ *nvj1*Δ *gdh1*Δ*gdh3*Δ*glt1*Δ *gdh1*Δ*gdh3*Δ *glt1*Δ *aat2*Δ			√	Reverse & Normal HPLC-Triple Quadrupole-MS	Targeted	POS and NEG

**Table 2:** The summary of data extracted from eligible articles. Abbreviations: Wt (Wildtype), AMP (Adenosine Monophosphate), IMP (Inosine monophosphate), GMP (Guanosine monophosphate), CMP (Cytidine monophosphate), UMP (uridine monophosphate) (>) indicates high levels of metabolites seen in one group in contrast to the other. (<) indicates, low levels of metabolites observed in one group in comparison to the other. (=) indicates comparable levels of metabolites demonstrated in both groups.

Reference	Metabolite of Interest	Notable Findings
[48]	5′AMP, 5′IMP, 5′GMP, 5′CMP, 5′UMP, adenosine, inosine, guanosine, uridine, cytidine, adenine, hypoxanthine, guanine, xanthine, uracil, cytosine, sedoheptulose-7-phosphate (S7P), sedoheptulose-1,7-bisphosphate (SBP)	**Carbon Starvation** Nucleosides, nucleobases (ratio of metabolite levels in deleted/Wt strain) Wt > *atg7*Δ (steady increase in inosine, guanosine, uridine, cytidine, hypoxanthine, guanine, xanthine, uracil levels in Wildtype than *atg7*Δ) Wt > *bcy*Δ (steady increase in adenosine, inosine, guanosine, uridine, cytidine, hypoxanthine, guanine, xanthine, uracil levels in Wildtype than *atg7*Δ) Wt > *snf*Δ (steady increase in adenosine, inosine, guanosine, uridine, cytidine, hypoxanthine, guanine, xanthine, uracil levels in Wildtype than *atg7*Δ) Wt > *pnp1*Δ (steady increase in hypoxanthine, guanine, xanthine, and uracil levels in Wildtype than *pnp1*Δ) Wt > *urh1*Δ (steady increase in hypoxanthine, guanine, xanthine, and uracil in Wildtype than *urh1*Δ Ribose-5-Phosphate and Sedoheptulose-7-phosphate (S7P) and Sedoheptulose-1,7-bisphosphate (SBP) species (ratio of metabolite levels in deleted/Wt strain) Wt > *atg7*Δ (steady increase in R5P, S7P and SBP in Wildtype than *atg7*Δ) Wt > *bcy*Δ (steady increase in R5P, S7P and SBP in Wildtype than *atg7*Δ) Wt > *snf*Δ (steady increase in S7P and SBP in Wildtype than *atg7*Δ, transient increase in R5P levels in Wildtype than *atg7*Δ) Wt > *pnp1*Δ (steady increase in R5P, S7P, SBP in Wildtype than *pnp1*Δ) Wt > *urh1*Δ (steady increase R5P, S7P, SBP in Wildtype than *urh1*Δ) Sedoheptulose-7-phosphate (S7P) and Sedoheptulose-1,7-bisphosphate (SBP) species (Intracellular concentration (uM)) Wt > *tkl1*Δ/*tkl2*Δ (steady increase in S7P and SBP in Wildtype than *tkl1*Δ/*tkl2*Δ strain) **Nitrogen Starvation:** Nucleosides, nucleobases sedoheptulose species (ratio of metabolite levels in deleted/Wt strain) Wt > *atg7*Δ (transient increase in inosine, guanosine, uridine, cytidine, hypoxanthine, guanine and uracil levels in Wildtype than *atg7*Δ) Wt = *atg7*Δ (comparable levels of adenosine and guanine between the two groups) **Phosphate Starvation** Nucleosides, nucleobases (ratio of metabolite levels in deleted/Wt strain) Wt = *atg7*Δ (comparable levels of adenosine, inosine, guanosine, uridine, cytidine, hypoxanthine, guanine, xanthine and uracil between the two groups)
[49]	5′AMP, 5′GMP, 5′CMP, 5′UMP, 3′AMP, 3′GMP, 3′UMP, Adenosine, Guanosine, Cytidine, Uridine, inosine, adenine, hypoxanthine, guanine, xanthine, uracil.	**Carbon Starvation** Nucleosides: adenosine, guanosine, cytidine, uridine (relative intensity) Wt > *atg2*Δ (high levels of nucleosides (except for adenosine) in Wildtype than *atg2*Δ) **Nitrogen Starvation** Intracellular Nucleosides: adenosine, guanosine, cytidine, uridine (µM) Wt > *atg2*Δ (transient increase in adenosine, guanosine, cytidine levels in Wildtype than *atg2*Δ) Wt > *atg2*Δ (steady increase in inosine and uridine in Wildtype than *atg2*Δ) Intracellular Nucleobases: adenine, hypoxanthine, guanine, xanthine, uracil (µM) Wt > *atg2*Δ (transient increase in adenosine, guanosine, cytidine levels in Wildtype than *atg2*Δ) Wt > *atg2*Δ (steady increase in inosine and uridine in Wildtype than *atg2*Δ) Extracellular Nucleosides: inosine, guanosine, cytidine, uridine (µM) Wt > *atg2*Δ (steady increase in inosine, guanosine, cytidine, and uridine in Wildtype than *atg2*Δ) Extracellular Nucleobases: xanthine, uracil (µM) Wt > *atg2*Δ (steady increase in xanthine and uracil in Wildtype than *atg2*Δ) Relative intensity levels of 3′AMP, 3′GMP, 3′UMP Wt > *atg2*Δ (Diminished levels of 3′AMP, 3′GMP and 3′UMP in *atg2*Δ strain than in Wildtype) Wt > *rny1*Δ (Diminished levels of 3′AMP, 3′GMP and 3′UMP in *atg2*Δ strain than in Wildtype) Relative intensity (Nucleosides and nucleobases) levels of guanosine, inosine, guanine, xanthine, cytidine, uridine, uracil Wt > *atg2*Δ (high levels of guanosine, inosine, guanine, xanthine, cytidine, uridine, uracil in Wildtype than *atg2*Δ) Wt < *pnp1*Δ (high levels of guanosine, inosine, cytidine, and uridine in *pnp1*Δ strain than in Wildtype) Wt = *pnp1*Δ (comparable levels of xanthine, guanine and uracil in *pnp1*Δ strain than in Wildtype) Wt < *urh1*Δ (high levels of cytidine and uridine in *urh1*Δ strain than in Wildtype) Wt < *urh1*Δ (high levels of uracil in Wildtype than in *urh1*Δ) Wt > *atg7*Δ & *atg17*Δ (high levels of adenosine, guanosine, cytidine, uridine in Wildtype than in *atg7*Δ & *atg17*Δ) Wt = *atg19*Δ, *atg32*Δ, *NVJ1*Δ, *atg11*Δ (comparable levels of adenosine, guanosine, cytidine and uridine)
[32]	Exploratory (Untargeted)	**Glucose Starvation** ATP, Glucose-6-phosphate (G6P), Ribose-5-phosphate (R5P), glutamate, sedoheptulose-7-guanine, phosphoenolpyruvate UDP-hexose Wt > *cbp2*Δ (High levels of ATP, Glucose-6-phosphate (G6P), Ribose-5-phosphate (R5P), glutamate, sedoheptulose-7-guanine, phosphoenolpyruvate UDP-hexose in Wildtype than in *cbp2*Δ Aspartate and glutamate Wt > *cbp2*Δ (High levels of aspartate and glutamate in Wildtype than in *cbp2*Δ) Acute Glucose Starvation: Hexose, Hexose-Phosphate, Pentose, NADH (sub-minute levels) *Non-Starved Wt* > *Starved Wt* (High levels of pentose phosphate, ribose phosphates and pentose in non-starved Wildtype than starved Wildtype) Acute Glucose Starvation: ATP, AMP (sub-minute) Non-Starved Wt > starved Wt cells (ATP) Starved Wt > non-starved Wt cells (AMP) Lipid Profiling Polyketides, Dolichols Starved Wt > non-starved Wt cells (High levels of Polyketides dan Dolichols in starved Wildtype than non-starved %ATP Wt = *atg14*Δ Wt > *pot1*Δ Wt > *atg2*Δ Wt > *atg2*Δ*pot1*Δ
[14]	Arginine, histidine, proline, methionine, isoleucine, (ratio of leucine/isoleucine), valine, serine, (ratio of threonine/histidine) phenylalanine, tyrosine, tryptophan, glutamine, glutamate, asparagine, aspartate, fructose-bisphosphate (FBP), 2/3-phosphoglycerate (2/3-PG), pyruvate, citrate, alpha-ketoglutarate (a-KG), succinate, fumarate, malate	**Nitrogen starvation** Amino acids, intermediates of glycolysis, and tricarboxylic acid (TCA) cycle (intracellular concentration (uM)) Wt = *atg1*Δ (comparable levels, however, aspartate, glutamate, glutamine, valine, and alpha-ketoglutarate were found to be slightly higher abundance in Wt than *atg1*Δ) Aspartate and Glutamate (relative abundance) Wt > *atg1*Δ, *atg6*Δ, *atg9*Δ, *atg15*Δ, *ypt7*Δ, *pep4prb1*Δ Wt = *atg19*Δ, *atg32*Δ, *NVJ1*Δ Wt > *gdh1*Δ*gdh3*Δ*glt1*Δ > *atg1*Δ (triple deletion reduces Glu and Asp by 50%) Wt > *glt1*Δ > *atg1*Δ Wt > *aat2*Δ (*aspartate levels completely abolished*)

## Data Availability

Not applicable.
